# Optimizing and forecasting the development of China’s sports industry structure

**DOI:** 10.1371/journal.pone.0328418

**Published:** 2025-10-31

**Authors:** Fanxiang Zhao, Hongjian Gao

**Affiliations:** 1 Department of Kinesiology, Yeungnam University, Gyeongsan, Korea; 2 Department of Physical Education, Kunsan National University, Gunsan, Korea; Xi'an University of Architecture and Technology, CHINA

## Abstract

The sports industry’s contribution to economic growth is increasingly pronounced, as structural optimization offers significant potential to enhance its impact on economic development. This study employs the grey relational analysis and GM (1,1) grey prediction models to examine the correlation between China’s sports sub-sectors and key national economic development indicators, providing projections for future trends. Results indicate that the sports manufacturing sector and related product sales demonstrate the strongest correlation with Gross Domestic Product (GDP), highlighting their critical role in fostering economic growth. The GM (1,1) model reliably forecasts future trends in the sports industry’s added value. Based on these insights, sector-specific recommendations are proposed to promote structural optimization and sustainable development in China’s sports industry.

## Introduction

With the rapid economic growth, China’s sports industry has witnessed substantial expansion, with its total scale increasing from 1,710.7 billion yuan in 2015 to 3,300.8 billion yuan in 2022 [1]. The rise in gross national product, per capita disposable income, and per capita consumption expenditure has further stimulated the development of the sports service industry. According to data released by the General Administration of Sport of China, the share of the sports service industry within the sports sector increased from 33.4% in 2015 to 57.3% in 2023, establishing it as the core industry of the sector.

The sports industry is recognized as an ‘industry of happiness,’ a green industry, and an emerging sector [2], plays a pivotal role in advancing high-quality economic development [3], meeting diverse public demands for sports, stimulating consumption, and driving economic growth [4]. Optimizing the sports industry structure can boost both the scale and quality of the sports service sector, facilitate the advancement of the sporting products industry, diversify and extend the industrial chain, and enhance the impact and driving force of the sports services sector [5]. Structural optimization of the sports industry amplifies its social and economic benefits, notably supporting initiatives like the Healthy China campaign, job creation, and rural revitalization [6]. Furthermore, responding to technological advancements and industrial transformation by promoting “Internet + Sports” development and fostering new business formats and models in the sports industry are essential directions for structural optimization [7].

While the sports industry has experienced remarkable expansion driven by rising GDP, increasing disposable income, and the growth of services, these quantitative gains have not been matched by qualitative improvements in industrial structure. The coexistence of rapid growth and persistent structural imbalances underscores a critical research problem: how to optimize the configuration and synergy of sub-sectors to ensure sustainable and high-quality development of the sports industry.

Currently, China’s sports industry faces challenges such as insufficient synergy among leading sectors, imbalances in the development of winter and summer sports resources, inadequate vertical integration within the industrial chain, and a relatively low level of modernization in the sports industry system [8]. Optimizing the structure of the sports industry is thus essential to overcoming these challenges and advancing high-quality development across the sector.

This study aims to employ grey system methods to quantify the relationship between China’s sports industry sub-sectors and economic growth, identify the areas with the greatest potential for future growth, and forecast the trends of value-added in the sports industry, thereby providing a scientific basis and policy pathways for the optimization of the industry structure.

Accordingly, this study addresses the following research questions:

1. What are the correlations between different sub-sectors of China’s sports industry and national economic indicators?
2. How are the interrelationships among sports industry sub-sectors structured, and what do they imply for the overall optimization of the industry?
3. How will China’s sports industry develop in the future, and what implications do these projections hold for sustainable and high-quality growth?

## Literature review

### Structural challenges and institutional context of China’s sports industry

Currently, China’s sports industry is undergoing domestic industrial upgrading amid complex international dynamics, transitioning from rapid growth to high-quality development. Existing studies on the structure of the sports industry generally agree on persistent structural challenges, such as imbalances between supply and demand, regional disparities, and gaps in the consumption structure [9]. The industry continues to face obstacles, including a low level of system modernization, a slow progression toward structural rationalization, an unsatisfactory external structure, and significant path dependence in industrial development [10].

Having emerged relatively recently, the Chinese sports industry still possesses largely untapped potential, and its contribution to economic growth has yet to be fully realized. Compared with the sports sectors of developed countries such as those in Europe, North America, South Korea, and Japan, the quality of development in China’s sports industry remains inadequate. Influenced by its sports management mechanisms, the industry relies heavily on a national system primarily oriented toward competitive sports, while the market economy remains underdeveloped [11].

When examining the static proportions of the sports services sector and the sports manufacturing sector, the structural evolution of China’s sports industry broadly aligns with the general patterns of industrialization. However, achieving structural optimization—consistent with high-quality economic and industrial development—requires moving beyond mere scale expansion and a narrow focus on sports services. This optimization should be guided by high-quality development objectives, with an emphasis on systematically understanding the non-linear structures and dynamic interactions within the subsystems of the sports industry. Sustainable development strategies must address these complexities and foster balanced growth with long-term potential [12].

In China, both the added value of the sports industry and the level of national economic development have risen steadily year after year, and the role of the sports industry within the national strategic framework—as well as its contribution to the overall economy—has become increasingly significant. Therefore, it is essential to further deepen systemic reforms, stimulate sports consumption, optimize the industrial structure, and advance high-quality development within the sports sector.

### Industrial structure optimization theory

The theory of industrial structure optimization has long been a central topic in development economics and industrial economics. It emphasizes that economic growth and sustainable development depend not only on the expansion of industrial scale but also on the rationalization and dynamic upgrading of industrial structures. From both theoretical and empirical perspectives, this view has been widely supported. For example, analyses based on provincial panel data in China reveal that investment in basic research significantly promotes both the rationalization and upgrading of industrial structures, with varying effects across different types of institutions [13].

Rationalization of industrial structure refers to the efficiency, coordination, and proportional balance among different industries or sub-sectors. A rationalized structure ensures that resources such as labor, capital, and technology are allocated efficiently, thereby reducing structural contradictions, avoiding redundancy, and enhancing overall productivity [14]. In the context of the sports industry, rationalization involves strengthening the synergy among sub-sectors such as sports manufacturing, services, and events, to achieve coordinated growth rather than isolated development [15].

Advancement of industrial structure emphasizes the upgrading of industries toward higher value-added, innovation-driven, and knowledge-intensive activities. This reflects the transition from labor-intensive to capital- and technology-intensive industries, and from basic production to diversified, service-oriented, and innovation-led development [16]. For the sports industry, advancement manifests in the shift from traditional manufacturing and competition-driven models to digital sports, sports technology, and health-oriented services, thereby meeting the diversified demands of consumers and contributing to high-quality development.

Industrial structure optimization theory also provides a policy-relevant framework for analyzing the pathways through which an industry can enhance its competitiveness and sustainability. It underscores the importance of structural adjustment, technological innovation, and integration with broader economic strategies. In China, the optimization of the sports industry is closely tied to national initiatives such as the “Healthy China” and “Sports Power” strategies, which highlight the role of structural transformation in achieving both economic and social development goals.

By adopting the perspective of industrial structure optimization, this study situates the sports industry not merely as an economic sector but as a dynamic system whose rationalization and advancement are critical for promoting high-quality and sustainable growth.

### Economic growth and industrial transformation of the sports industry

As a key driver of global economic growth, the strategic importance of the sports industry has been widely acknowledged. Milano and Chelladurai [17], in their seminal research on the U.S. sports industry, emphasized its role in underpinning the national economy, and subsequent studies have generally reinforced this perspective. However, such macro-level analyses often treat the sports industry as a homogeneous sector, overlooking the heterogeneity across subsectors and the unevenness of regional development. Building on this foundation, Zhang et al. [18] proposed the “double-cycle effect” of mega international sporting events such as the World Cup and the Olympics, arguing that they can generate both short-term boosts in tourism and consumption and long-term urban development through infrastructure investment. While this perspective is valuable in highlighting the multi-layered impact of events, it risks overemphasizing positive spillovers, as numerous cases also show event-driven debts, underutilized facilities, and social displacement, suggesting that the “legacy” of mega-events is far more contested than often assumed.

In the context of new-type urbanization, Su et al. [19] argued that the collaborative development of the sports industry and urban-cluster economies has become a crucial pathway for regional transformation. This line of research advances the discussion by integrating sports with broader processes of urban and regional planning. Yet, empirical validation of such synergies remains limited, particularly regarding how sports development interacts with uneven regional growth trajectories. Similarly, Hao and Kong [20] emphasized the sports industry’s potential to optimize labor markets and investment structures, but their work largely remains at a conceptual level, and more rigorous quantitative analyses are needed to substantiate such claims.

A substantial body of scholarship has further highlighted the agglomeration development of sports industry clusters as a key pathway to enhancing regional efficiency. Cong and Wang [21] demonstrated that industrial clusters, through resource integration and industrial chain coordination, can significantly amplify agglomeration effects, fostering employment and stimulating related industries such as tourism and catering. While these findings underscore the catalytic role of clusters, they often assume favorable institutional conditions and may underestimate potential risks such as over-concentration, resource competition, and regional inequality. The case of Ukraine’s fitness industry [22] provides empirical support for cluster-based growth, but its transferability to contexts with different institutional and cultural conditions, such as China, remains uncertain. To address structural rigidities, Zhu and Zhao [23] proposed a model of structural optimization, stressing the importance of breaking down industry barriers and enhancing factor mobility. This is a critical contribution, yet questions remain regarding how such measures can be operationalized in practice, especially within sports systems that are heavily shaped by state intervention and entrenched path dependencies.

Collectively, existing studies affirm the economic significance of the sports industry through pathways such as event economies, urban integration, and industrial clustering. Nonetheless, the literature tends to privilege positive externalities while neglecting contradictions such as underutilization of infrastructure, uneven regional benefits, and the tension between state-driven objectives and market-based mechanisms.

### Technological empowerment and green transformation of the sports industry

Digital technologies are increasingly reshaping the ecological landscape of the sports industry. Existing research highlights a wide range of potential benefits: big data technologies are said to enhance event operation efficiency, optimize user experiences, and support precise decision-making [24]; cloud computing platforms are argued to solve the problem of fragmented data sharing [25]; and Lv et al. [26] identify blockchain as a promising frontier for future industry innovation. Gruettner [27] further emphasizes that stakeholders must engage in closer collaboration to unleash these innovative potentials. Moreover, scholars suggest that the integration of the digital economy and the sports industry can stimulate sports consumption, drive industrial transformation, and improve efficiency [28]. While these contributions underscore the transformative potential of digital technologies, they often adopt a techno-optimistic stance, focusing on opportunities while neglecting structural challenges such as digital divides, uneven technological adoption between urban and rural areas, data privacy and security concerns, and the limited absorptive capacity of small- and medium-sized enterprises in the sports sector [29,30]. Without addressing these issues, the promised gains from digital transformation may be realized only unevenly, reinforcing rather than reducing disparities across regions and market actors.

Parallel to the technological turn, the green development trajectory of the sports industry has become a focal point of inquiry. Millington et al. [31] argue that sports programs can offset the environmental externalities of traditional extractive industries through ecological restoration and community participation, thereby contributing to sustainable development. Scholars also highlight the sector’s potential role in fostering social inclusion and gender equality [32], with investments in public sports facilities lowering participation barriers, enhancing community health, and indirectly reducing healthcare costs through a “health economy.” Su et al. [19] similarly emphasize the synergy between sports industry development and urban-cluster economies, suggesting that coordinated strategies can optimize resource allocation and improve living environments. Nevertheless, the literature tends to idealize the social and ecological benefits of sports while underexploring their trade-offs. For example, large-scale venue construction and mega-event hosting often generate high carbon emissions, land-use conflicts [33], and social displacement, raising questions about whether green outcomes are truly realized in practice [34]. Research indicates that mega sporting events—from transportation and infrastructure development to venue operations—are significant sources of greenhouse gas emissions [35]. Moreover, empirical evidence on how sports participation contributes to long-term health cost savings remains fragmented and context-specific. This indicates a need for more rigorous longitudinal and comparative studies that critically assess both the promises and contradictions of the sports industry’s green transformation.

In the context of globalization, scholars have examined the transnational integration of resources in the sports industry, noting both opportunities and challenges. On one hand, globalization enables knowledge spillovers and capital flows, facilitating the catch-up of latecomer regions through technological transfer and human capital accumulation [36]. On the other hand, cultural homogenization, regional disparities, and unequal distribution of benefits remain pressing issues [37]. Huang [38] proposes a “regional gradient development strategy,” suggesting differentiated paths: eastern regions should prioritize innovation and high-end services, while central and western regions should focus on factor integration and industrial clustering. This framework provides a valuable corrective to one-size-fits-all globalization narratives, yet it risks underestimating the structural constraints imposed by global competition, such as dependency on foreign technologies and capital volatility. Additionally, existing studies rarely explore how domestic institutional arrangements—particularly the interplay between state intervention and market forces—mediate the uneven impacts of globalization on the sports industry.

Taken together, the literature highlights the transformative promise of digitalization, green development, and globalization for the sports industry, but it often privileges opportunity-driven narratives while overlooking contradictions, uneven effects, and context-specific limitations.

## Method

### Grey correlation analysis

Grey correlation analysis is a quantitative research method within grey system theory. Its basic principle is to assess the closeness of the relationship between different sequences by evaluating the similarity of the geometric shapes of their curves. The closer the shapes of the broken lines, the higher the degree of correlation between the corresponding sequences, and vice versa. Through specific data processing of random factor sequences, grey correlation analysis identifies the degree of relevance among factors, highlights the primary contradictions, and reveals the main characteristics and influencing factors [39].

First, the data must first undergo dimensionless processing to standardize values for analysis. This paper employs the initial value method for calculation, as outlined below.


$$x_{ij}=x_{ij}\;/\;x_{\text{1}j}$$ (1)

Here, ***j*** represents the indicator, and ***i*** represents the year. Next, the correlation coefficient is calculated as follows.


$$\zeta_{i}(k)=\frac{\underset{i}{min}\underset{k}{min}|x_{\text{0}}(k)-x_{i}(k)|+\rho \cdot \underset{i}{max}\underset{k}{max}|x_{\text{0}}(k)-x_{i}(k)|}{\left|x_{\text{0}}(k)-x_{i}(k)|+\rho \cdot \underset{i}{max}\underset{k}{max}|x_{\text{0}}(k)-x_{i}(k)\right|}$$ (2)

Finally, the mean of the correlation coefficients for each indicator is calculated to obtain the degree of association for that indicator.

### Grey prediction model GM (1, 1)

Grey prediction is an effective approach for forecasting in systems characterized by uncertainty, incomplete information, or limited data samples. It evaluates the similarities and differences in development trends among factors within the system (grey relational analysis) and processes the original data using methods such as the Accumulated Generating Operation, which reduces randomness and enhances data regularity. Based on the processed data sequence, a first-order univariate differential equation model, GM (1,1), is then constructed to predict future development trends [40].

The GM (1,1) model has relatively modest requirements regarding the amount of data and does not impose strict assumptions about data distribution, which makes it particularly effective when the data are limited or of low quality [41]. By contrast, the ARIMA model usually requires a large volume of historical data for model fitting and verification, while regression analysis depends on assumptions such as normality and linearity. When these assumptions are not satisfied, the reliability of the results is compromised. Moreover, in many practical application scenarios [42], the GM (1,1) model has demonstrated high prediction accuracy, particularly in medium- and short-term forecasting.

In summary, the choice of the GM (1,1) model as the primary analytical tool for this study reflects a comprehensive consideration of factors such as data characteristics, the complexity of the research problem, and the inherent advantages of the model itself. The detailed calculation method is presented below.

(1) Generate the accumulated sequence ***x***^(1)^


$$x^{\left(\text{1}\right)}(k)=\sum_{i=\text{1}}^{k}x^{\left(\text{0}\right)}(i),\;k=\text{1},\text{2},\ldots ,n$$ (3)

(2) Generate the adjacent mean equal-weight sequence ***z***^(1)^ of ***x***^(1)^


$$z^{\left(\text{1}\right)}(k)=\frac{\text{1}}{\text{2}}\left(x^{\left(\text{1}\right)}(k)+x^{\left(\text{1}\right)}(k-\text{1})\right),\;k=\text{2},\text{3},\ldots ,n$$ (4)

(3) Establish the GM (1,1) model


$$x^{\left(\text{0}\right)}(k)+az^{\left(\text{1}\right)}(k)=b,\;k=\text{2},\text{3},\ldots ,n$$ (5)

$$\frac{dX^{\left(\text{1}\right)}}{dt}+aX^{\left(\text{1}\right)}=b$$ (6)

Here, ***a*** and ***b*** are parameters to be determined.

(4) Solve for ***a*** and ***b*** using the least squares method


$$(a,b{)}^{T}={\left(B^{T}B\right)}^{-\text{1}}B^{T}Y$$ (7)

Define matrices ***B*** and ***Y*** as follow:


$$B=[\begin{array}{cc} -z^{\left(\text{1}\right)}(\text{2}) & \text{1}\\ -z^{\left(\text{1}\right)}(\text{3}) & \text{1}\\ \vdots & \vdots \\ -z^{\left(\text{1}\right)}(n) & \text{1} \end{array}],\;Y=\left[\begin{array}{c} x^{\left(\text{0}\right)}(\text{2})\\ x^{\left(\text{0}\right)}(\text{3})\\ \vdots \\ x^{\left(\text{0}\right)}(n) \end{array}\right]$$ (8)

(5) After solving for ***a*** and ***b***, obtain the time response formula


$${\hat{x}}^{\left(\text{1}\right)}(k+\text{1})=\left[x^{\left(\text{0}\right)}(\text{1})-\frac{b}{a}\right]e^{-ak}+\frac{b}{a},\;k=\text{0},\text{1},\ldots ,n-\text{1}$$ (9)

(6) Perform inverse accumulation to obtain the predicted values of the original data sequence


$${\hat{x}}^{\left(\text{0}\right)}(k+\text{1})={\hat{x}}^{\left(\text{1}\right)}(k+\text{1})-{\hat{x}}^{\left(\text{1}\right)}(k)=\left(\text{1}-e^{-a}\right)\left[x^{\left(\text{0}\right)}(\text{1})-\frac{b}{a}\right]e^{-ak}$$ (10)

(7) Calculate the model’s absolute and relative errors


$$\varepsilon^{\left(\text{0}\right)}(i)=x^{\left(\text{0}\right)}(i)-{\hat{x}}^{\left(\text{0}\right)}(i)$$ (11)

$$\omega^{\left(\text{0}\right)}(i)=\left|\frac{x^{\left(\text{0}\right)}(i)-{\hat{x}}^{\left(\text{0}\right)}(i)}{x^{\left(\text{0}\right)}(i)}\right|$$ (12)

(8) Conduct model accuracy verification. The calculation process is as follows.


$${\bar{x}}^{\left(\text{0}\right)}=\frac{\text{1}}{n}\sum_{i=\text{1}}^{n}x^{\left(\text{0}\right)}(i)$$ (13)

$$S_{\text{1}}=\sqrt{\frac{\text{1}}{n-\text{1}}\sum_{i=\text{1}}^{n}{\left(x^{\left(\text{0}\right)}(i)-{\bar{x}}^{\left(\text{0}\right)}\right)}^{\text{2}}}$$ (14)

$$\Delta^{\left(\text{0}\right)}(i)=x^{\left(\text{0}\right)}(i)-{\hat{x}}^{\left(\text{0}\right)}(i)$$ (15)

$${\bar{\Delta }}^{\left(\text{0}\right)}=\frac{\text{1}}{n}\sum_{i=\text{1}}^{n}\Delta^{\left(\text{0}\right)}(i)$$ (16)

$$S_{\text{2}}=\sqrt{\frac{\text{1}}{n-\text{1}}\sum_{i=\text{1}}^{n}{\left(\Delta^{\left(\text{0}\right)}(i)-{\bar{\Delta }}^{\left(\text{0}\right)}\right)}^{\text{2}}}$$ (17)

$$C=\frac{S_{\text{1}}}{S_{\text{2}}}$$ (18)

$$p=P\left\{\left|\Delta^{\left(\text{0}\right)}(i)-{\bar{\Delta }}^{\left(\text{0}\right)}\right|<\text{0}.\text{6}\text{7}\text{4}\text{5}S_{\text{1}}\right\}$$ (19)

Finally, determine the model accuracy based on the calculated values of ***C*** and ***p***.

The GM (1,1) model is appropriate for capturing short-term dynamics in relatively stable systems. However, extraordinary disruptions such as the COVID-19 pandemic (2020–2022) may affect the stability of the model’s assumptions.

### Data sources

The added value of the sports industry and the added value of each sub-industry of the sports industry are derived from the Announcement of the Total Scale and Added Value Data of National Sports Industry from 2015 to 2023 by the State Sports General Administration of China https://www.stats.gov.cn/sj/zxfb/202312/t20231229_1946084.html (accessed on August 5, 2024); The data of GDP and per capita GDP come from the Announcement on Final Verification of GDP from 2015 to 2023 issued by the National Bureau of Statistics of China https://www.stats.gov.cn/sj/zxfb/202312/t20231229_1946058.html (accessed on August 8, 2024); The data of per capita disposable income and per capita consumption expenditure come from the National Bureau of Statistics of China’s ‘China Statistical Yearbook’ https://www.stats.gov.cn/sj/ndsj/ (accessed on August 12, 2024). All data are recorded at current-year prices. The raw and processed data underlying the findings are available in Appendix.

## Results

### Selection of comparison sequences

According to the classification of sports industry in China’s Announcement on the Total Scale and Added Value of National Sports Industry, it is ranked as follows: sports management activities (X1), sports competition and performance activities (X2), sports fitness and leisure activities (X3), sports venues and facilities management (X4), sports brokerage and agency, advertising and exhibition, performance and design services (X5), sports education and training (X6), sports media and information services (X7), sales, rental and trade agency of sporting goods and related products (X8), other sports services (X9), manufacturing of sporting goods and related products (X10), and construction of sports venues and facilities (X11). The added value of each sub-sector of sports industry from 2015 to 2023 is selected as the comparison sequence, which lasts for 8 years and meets the requirement of minimum sample size of grey correlation analysis for 4 years. See Table 1 for details.

**Table 1:** Original data table of added value of sports subdivision industry (100 million yuan).

No.	2015	2016	2017	2018	2019	2020	2021	2022	2023
X1	115	143.8	262.6	390	451.9	459	515	599	689
X2	52.6	65.5	91.2	103	122.3	103	129	145	300
X3	129.4	172.9	254.9	477	831.9	736	892	962	1378
X4	458.1	567.6	678.2	855	1012.2	808	1031	1106	1289
X5	14	17.8	24.6	106	117.8	98	119	113	239
X6	191.8	230.6	266.5	1425	1524.9	1612	1795	1888	2278
X7	40.8	44.1	57.7	230	285.1	339	406	452	502
X8	1562.4	2138.7	2615.8	2327	2562	2574	2955	3119	3159
X9	139.6	179.7	197.2	616	707	645	733	796	1015
X10	2755.5	2863.9	3264.6	3399	3421	3144	3433	3686	3832
X11	35.3	50.3	97.8	150	211.9	217	236	226	234

### Selection of reference sequences

If want to study the relationship between China’s sports subdivision industry and national economy through grey correlation analysis, a national economic index should be determined as a reference sequence. Usually, per capita disposable income, per capita consumption expenditure, gross domestic product (GDP) and per capita GDP are used to measure the development of national economy. In order to determine the representative indexes, this paper uses grey correlation analysis to calculate the grey correlation degree between the added value of sports industry in China and GDP, per capita GDP, per capita disposable income and per capita consumption expenditure from 2015 to 2023. The highest correlation degree between the four national economic indexes and the added value of sports industry represents the closest relationship between the index and the added value of sports industry, and it is the most representative to choose this index as the research on the correlation degree between sports subdivision industry and national economy. The calculation results are shown in Table 2.

**Table 2:** Grey correlation degree between added value of sports industry and national economic indexes.

Category	Grey correlation degree	Sort
Added value of sports industry and GDP	0.658199377	1
Added value of sports industry and per capita GDP	0.627344976	3
Added value of sports industry and per capita disposable income	0.627921497	2
Added value of sports industry and per capita consumption expenditure	0.565824987	4

According to Table 2, the grey correlation degree between the added value of sports industry and GDP is the highest, which shows that there is a strong correlation between the added value of sports industry and GDP, and it is representative to use GDP as an inspection index for the correlation analysis between sports industry and national economy.

### Grey correlation degree between added value of sports subdivision industry and GDP

The average value of correlation coefficient is the grey correlation degree between sports subdivision industry and GDP, and the calculation results are shown in Table 3.

**Table 3:** Grey correlation degree between added value of sports subdivision industry and GDP.

Sports subdivision industry	Correlation degree	Sort
Sports management activities	0.8011	5
Sports competition and performance activities	0.9027	4
Sports fitness and leisure activities	0.7240	8
Management of sports venues and facilities	0.9425	3
Sports brokerage and agency, advertising and exhibition, performance and design services	0.6627	11
Sports education and training	0.6657	10
Sports media and information service	0.6740	9
Sales, rental and trade agency of sporting goods and related products	0.9722	2
Other sports services	0.7738	6
Manufacturing of sporting goods and related products	0.9730	1
Construction of sports venues and facilities	0.7323	7

According to Table 3, the grey correlation degree between the added value of sports subdivision industries and GDP can be divided into three levels.

Level 1: Manufacturing of sporting goods and related products, sales, rental and trade agency of sporting goods and related products, management of sports venues and facilities, Sports competition and performance activities, and Sports management activities, all of which have a correlation degree greater than 0.8 with the national economy. It suggests that the sports industry has already been deeply embedded in the national economic system, forming close linkages with industrial production, service provision, and market operations. At the same time, this phenomenon highlights the structural feature that the development of the sports industry still relies heavily on traditional, tangible, and management-intensive sectors, raising the question of how to further enhance the value-added contribution from emerging fields such as sports technology, digital platforms, and cultural-creative integration.

Level 2: Construction of sports venues and facilities, sports fitness and leisure activities, other sports services, all of which have a correlation degree greater than 0.7 with the national economy. This phenomenon reflects the growing importance of public consumption and service-oriented segments in driving sports industry development. It indicates that the sports industry is increasingly shifting from a production-oriented model toward a consumption- and experience-oriented model, in which infrastructure construction and leisure participation play a pivotal role. At the same time, this also reveals a structural issue: although these sectors have shown steady growth, their overall contribution to economic integration remains secondary compared with the core sectors identified in Level 1.

Level 3: Sports media and information service, sports education and training, sports brokerage and agency, advertising and exhibition, performance and design services, all of which have a correlation degree less than 0.7 with the national economy. This phenomenon indicates that these emerging and service-oriented sectors, though essential for the long-term modernization and diversification of the sports industry, have not yet formed strong and stable linkages with the broader economic system. It reflects a structural problem that the high value-added and knowledge-intensive segments of the sports industry remain underdeveloped, limiting their potential to drive economic growth at scale.

### Grey correlation analysis between sports subdivision industries

Calculate the grey correlation degree between sports subdivision industries and form a grey correlation degree matrix, as shown in Table 4. If each horizontal or vertical value of an industry is greater than the corresponding horizontal or vertical value of other industries, it means that the industry is the strongest related industry. If each horizontal or vertical value of an industry is less than the corresponding horizontal or vertical value of other industries, it means that the industry is the weakest related industry.

**Table 4:** Grey correlation degree matrix of sports subdivision industry.

	X1	X2	X3	X4	X5	X6	X7	X8	X9	X10	X11
X1	1	0.74	0.76	0.72	0.63	0.59	0.61	0.69	0.84	0.65	0.75
X2	0.79	1	0.70	0.97	0.64	0.61	0.62	0.92	0.74	0.86	0.68
X3	0.77	0.65	1	0.64	0.77	0.69	0.71	0.63	0.80	0.59	0.88
X4	0.78	0.97	0.70	1	0.63	0.62	0.63	0.94	0.75	0.88	0.68
X5	0.67	0.62	0.79	0.61	1	0.87	0.78	0.60	0.71	0.57	0.75
X6	0.66	0.62	0.75	0.62	0.88	1	0.89	0.60	0.71	0.60	0.72
X7	0.70	0.65	0.79	0.65	0.82	0.90	1	0.63	0.75	0.63	0.77
X8	0.76	0.93	0.69	0.95	0.64	0.61	0.62	1	0.73	0.92	0.68
X9	0.83	0.67	0.78	0.67	0.66	0.64	0.67	0.64	1	0.62	0.80
X10	0.74	0.87	0.67	0.90	0.62	0.62	0.63	0.92	0.72	1	0.66
X11	0.74	0.61	0.87	0.59	0.71	0.64	0.67	0.59	0.80	0.55	1

Table 4 shows that there are neither the strongest nor the weakest related industries among all sub-sectors, indicating that mature leading industries have not yet emerged within the sports industry [39]. A comparison of the data reveals that the horizontal and vertical correlation coefficients among X2, X4, and X8 all exceed 0.9, while those between X8 and X10 are also greater than 0.9. This suggests that sports competition and performance activities, management of sports venues and facilities, and the sales, rental, and trade agency of sporting goods and related products are the most closely related. Furthermore, the manufacturing of sporting goods and related products demonstrates a strong correlation with the sales, rental, and trade agency of sporting goods and related products.

The high correlation among these sub-sectors may be explained by their interdependent roles within the sports industry value chain. For example, manufacturing of sporting products provides the goods necessary for sales and rental activities, which in turn support venue operations and the hosting of sports competitions and performances. This vertical integration implies that fluctuations in one sub-sector can directly influence the others, resulting in strong grey correlation coefficients. Furthermore, these industries are often driven by shared market demand and policy incentives, which reinforces their synchronous growth patterns. The observed correlations reflect the structural interconnections of key commercial and operational activities in the sports sector, highlighting how core industries are closely linked even in the absence of fully mature leading sub-sectors.

### Forecast of the added value of the sports industry

The GM (1,1) model was applied to the added value data of the sports industry from 2015 to 2023. Initial findings indicated that the raw data did not satisfy the level ratio test; however, after shifting by 1251, the data met the test criteria. Therefore, GM (1,1) predictions were conducted using the shifted data, and the prediction results were adjusted by subtracting 1251 before conducting the error test. The calculated ***C*** value was 0.2325, and the ***p*** value was 1. According to Table 5, this model demonstrates good accuracy.

**Table 5:** Model accuracy standards.

Grade	*p*	*C*
Good	***p*** ≥ 0.95	***C*** ≤ 0.35
Qualified	0.80 ≤ ***p*** < 0.95	0.35 < ***C*** ≤ 0.50
Average	0.70 ≤ ***p*** < 0.80	0.50 < ***C*** ≤ 0.65
Unqualified	***p*** < 0.70	***C*** > 0.65

**Fig 1:**
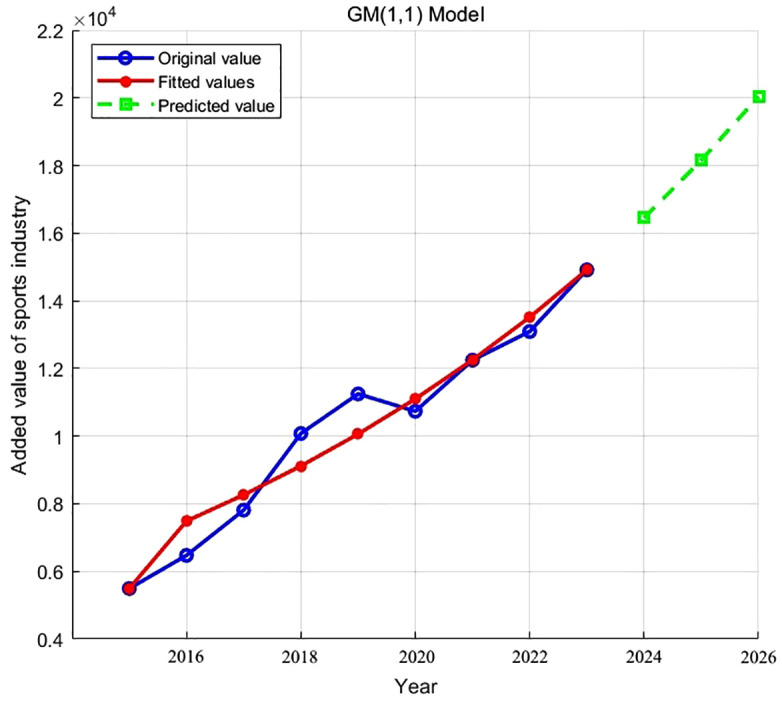
Forecast of added value of sports industry.

The forecast of the added value of the sports industry for the next three years is shown in Fig 1. As seen in Table 6, the average relative error of the prediction model is 5.36%, which is less than 10%, indicating the model’s accuracy.

**Table 6:** Grey forecast detailed data.

Year	Original value	Predicted value	Residual	Relative error	Average relative error
2015	5494.4	5494.40	0	0	5.36%
2016	6474.8	7488.38	1013.58	15.65	
2017	7811.4	8262.90	451.50	5.78	
2018	10078	9117.53	−960.47	9.53	
2019	11248.1	10060.55	−1187.55	10.56	
2020	10735	11101.11	366.11	3.41	
2021	12245	12249.30	4.30	0.04	
2022	13092	13516.24	424.24	3.24	
2023	14915	14914.22	−0.78	0.01	
2024		16456.80			
2025		18158.92			
2026		20037.10			

The predicted values indicate a continuous upward trend in the sports industry, projecting growth from 14,915 billion yuan in 2023 to 20,037.10 billion yuan in 2026. Minor deviations between predicted and actual values in 2016, 2018, 2019, and 2022 suggest that short-term disruptions, such as economic fluctuations or external shocks like the COVID-19 pandemic, may temporarily affect industry performance. Overall, the model captures the general trajectory of growth and provides useful insights into future trends of the sports sector.

## Discussion

### Detailed analysis of the correlation between sports subdivision industry and national economy

#### Manufacturing of sporting goods and related products.

The manufacturing of sporting goods and related products exhibits the highest correlation with GDP (0.9730), underscoring its central role in driving economic growth. Although economic development has supported the expansion of this sector, the proportion of manufacturing value added in the total sports industry declined from 50.2% in 2015 to 25.7% in 2023. Despite this decline, manufacturing output still accounted for 40.0% of the industry’s total output in 2023, indicating that the sector remains a core pillar of the sports industry.

This downward trend in relative contribution can be attributed to structural shifts in global production. Major international brands, such as Nike and Adidas, have relocated part of their production to countries like Vietnam, thereby reducing China’s export share in sports manufacturing. Nevertheless, strong domestic and international demand continues to sustain growth, suggesting resilience in the sector despite external pressures.

China’s sports manufacturing has gradually evolved from a low-end processing and export-oriented model to a mid-level position in the industrial chain, integrating design, R&D, production, and sales. Leading companies have expanded into international markets through targeted marketing strategies, reflecting a transition from “quantity” to “quality” and from low value-added to higher value-added production [43].

However, challenges remain. The sports equipment sector lacks well-established domestic brands and core technological competitiveness [44], particularly in niche areas such as rock climbing, surfing, and sailing. High-tech R&D and high-value-added products are still limited, with many high-end training and measurement devices dependent on imports. These patterns indicate that, while the sector continues to be a traditionally advantageous segment of the sports industry, there is a need for strategic enhancements: refining production models, fostering core technologies, innovating marketing approaches, and improving product competitiveness. Addressing these gaps is crucial for sustaining long-term growth and strengthening China’s position in the global sports manufacturing value chain.

#### Sales, rental and trade agency of sporting goods and related products.

The sales, rental, and trade agency of sporting goods and related products exhibits the second-highest correlation with GDP (0.9722), underscoring its critical role in promoting economic growth. In 2023, this sub-sector accounted for 14.8% of the sports industry’s total output, ranking first within the sports services sector. The strong correlation reflects the growing consumption of sporting goods, driven by rapid economic development, rising income levels, and increasing purchasing power, which position physical consumption as the dominant form of sports-related expenditure [45].

The steady expansion of the sports retail industry can be attributed to both rising consumer demand and technological advancements that have enhanced labor productivity. However, several structural and market constraints continue to limit the sector’s performance. The integration of online and offline sales channels remains insufficient, consumption maturity is relatively low, and the variety of products and services is inadequate [46,47]. These factors collectively constrain the transformation and long-term growth of the sports retail industry.

To address these challenges and enhance long-term development, companies need to diversify product offerings, expand market supply, optimize service quality, and establish fully integrated online and offline sales channels that enhance consumers’ hedonic and social value. In addition, government policies play a pivotal role in supporting this sector [48]. Active promotion of the integration of sports with related industries—such as culture, elderly care, exhibitions, media, finance, and technology—can leverage regional advantages. Maximizing the impact of government funding and encouraging social investment will further facilitate the transformation and sustained growth of the sporting products sales, rental, and trade agency industry, ensuring that it continues to contribute significantly to the broader sports economy.

#### Management of sports venues and facilities.

The management of sports venues and facilities exhibits a high correlation with GDP (0.9425), indicating a significant relationship between effective facility management and the broader economy. Well-maintained and efficiently operated venues enhance residents’ sporting experiences, foster greater participation, and stimulate sports-related consumption [49]. This strong correlation reflects not only the direct economic contributions of venue operations but also the indirect benefits of promoting an active lifestyle among the population.

Policy initiatives have played a crucial role in shaping this sub-sector. The 2015 ‘Measures for the Operation and Management of Stadiums and Gymnasiums’, issued by the General Administration of Sport of China, emphasized prioritizing sports functions, improving public services, expanding service scope, elevating service quality, and enhancing operational efficiency. These guidelines facilitated a transition toward sports-centered and diversified management approaches, contributing to the sector’s sustained growth.

Furthermore, the development of sports service complexes and industry clusters around stadiums and gymnasiums—tailored to local economic, social, and urban development needs—has created synergies with sports events, tourism, performances, leisure, and commerce [50]. This integrated model not only optimizes the utilization of existing infrastructure and minimizes resource waste but also enriches the cultural and recreational lives of local residents.

The surge in residents’ sports participation, particularly in fitness and health-related activities such as home exercise programs, live-streamed workouts, and traditional practices like Ba Duan Jin and Tai Chi Chuan, reflects a broader societal trend toward active lifestyles and health preservation. These patterns indicate that the sports venue and facility management industry is closely intertwined with economic development and rising living standards, suggesting that continued investment in facility quality, diversified services, and community-oriented programs will be critical for sustaining long-term growth and maximizing the sector’s social and economic contributions.

#### Sports competition and performance activities.

The sports competition and performance industry exhibits a high correlation with GDP (0.9027), reflecting its substantial role in contributing to economic growth. Despite this strong relationship, the domestic scale of the industry remains relatively limited, partly due to the high financial requirements for team establishment and management, venue operation and maintenance, athlete training and rehabilitation, and event planning and promotion. Additionally, in economically underdeveloped regions, mass sports consumption is low, concentrating competition activities primarily in economically advanced first-tier and emerging first-tier cities. These patterns suggest that economic conditions and regional disparities strongly influence the distribution and scale of sports competitions and performances.

Policy initiatives have aimed to accelerate the development of this industry. The 2018 ‘Guiding Opinions on Accelerating the Development of the Sports Competition and Performance Industry’, issued by the General Office of the State Council, sets a target scale of 2 trillion yuan by 2025 and emphasizes the creation of a system characterized by diverse products, a rational structure, a solid foundation, and balanced development. The plan includes developing influential sports event cities and industry clusters, launching high-quality branded events, establishing sports competition and performance brands with independent intellectual property rights, and fostering market-competitive enterprises.

The industry has emerged as a key driver of sustainable economic and social development. Strategic development requires focusing on professional leagues in basketball, football, and volleyball, enhancing league quality and appeal, supporting niche sports, and establishing a well-structured league hierarchy. Hosting major international events [51] leverages broader economic and social benefits and integrates international sports resources, while promoting amateur leagues [52] and encouraging private organizations to conduct community competitions supports grassroots participation. These initiatives indicate that the sports competition and performance industry has the potential to unlock sports consumption, stimulate related sectors, and serve as a new growth engine within China’s broader sports industry.

#### Other sub-sectors.

The correlation between other sub-sectors and GDP is below 0.9, indicating a relatively weaker linkage with overall economic growth. In 2023, the sports education and training and the sports fitness and leisure activities contributed 7.8% and 7.5% of the total output of the sports industry, respectively, reflecting notable economic presence. However, their grey correlation with GDP in terms of added value is only 0.6657 and 0.7240, suggesting a weakening positive feedback loop between public demand for health and leisure activities and macroeconomic growth. This trend may be closely associated with demographic changes in China, as shifting population structures may limit the short-term impact on structural upgrading within the sports industry [53].

To strengthen the role of these sub-sectors and promote sustainable growth, targeted strategies are required. First, integration of sports and education should be enhanced by leveraging retired athletes and social resources to support campus sports through government-funded service programs. This approach can improve sports competitions in schools and provide after-school sports guidance, thereby fostering the development of the physical education and training industry. Standardizing the sports training market [54] and emphasizing children’s physical fitness and youth sports training will help establish a robust foundation for long-term industry growth.

Second, emerging sub-sectors such as sports brokerage and agency, advertising and exhibition services, performance and design services, and sports media and information services require further development. Leveraging digital platforms, news media, and the Internet [55], these sectors can expand market reach and improve service quality. Establishing industry standards and fostering professional talent through partnerships among universities, sports brokerage firms, and media companies will strengthen the sports economy and sports media industries, facilitating better integration with core sports sectors. Collectively, these measures can enhance the contribution of underdeveloped sub-sectors to the broader sports industry, optimize resource allocation, and promote sustainable and diversified industry growth.

### Detailed analysis between sports subdivision industries

The correlations among sports sub-sectors suggest that as China enters a new development phase, the high-quality advancement of the sports industry should prioritize the manufacturing, retail, and service sectors. Guided by the principles of “innovation,” “integration,” and “coordination,” it is essential to strengthen the leading role of the sports manufacturing sector while simultaneously aligning the development of the sporting products retail industry with evolving consumer demands and technological advancements. The manufacturing sector not only drives economic growth but also provides the essential supply and innovation foundation for downstream retail and service activities, underscoring its strategic significance within the broader industry.

The sports competition and performance industry functions as the core and foundation of the entire sports sector. Accelerating the upgrade of this sub-sector is critical for fostering high-quality development [38]. This requires improving the professionalism, structure, and appeal of competitive leagues, promoting niche sports, and supporting both international and community-level competitions. By enhancing the organization and branding of sports events, the industry can attract investment, expand audience engagement, and stimulate related sub-sectors, including sports media, sports brokerage, and leisure services.

Furthermore, fostering synergies across sub-sectors—such as sports media, sports brokerage, leisure sports, venue management, and competition performance activities—can amplify the reach and economic impact of sporting events. Strategic integration may include leveraging digital media platforms to enhance event visibility, combining venue management with recreational and commercial services to optimize resource utilization, and aligning marketing efforts across retail, media, and competition sectors. These coordinated efforts will not only enhance the economic contribution of the sports industry but also support broader social objectives, including increased participation in physical activity, youth development, and regional economic growth. By adopting a holistic, cross-sectoral development strategy, China’s sports industry can achieve sustainable, high-quality advancement while fostering innovation and competitiveness across all sub-sectors.

### Sports industry development forecast

The forecasting results derived from the GM (1,1) model indicate that China’s sports industry will continue to expand steadily in the coming years, with its added value expected to reach nearly 20 trillion yuan by 2026. While these projections demonstrate the growth potential of the industry, their significance can be better understood through the lens of industrial structure optimization theory.

First, the rationalization dimension suggests that future growth will depend not only on scale expansion but also on the improved coordination among sub-sectors. The forecasted upward trajectory reflects an opportunity to address existing imbalances, such as the over-reliance on manufacturing and the underdevelopment of service-oriented segments like fitness, education, and digital sports [56]. Rationalizing the structure requires enhancing synergies across sub-sectors, which can generate multiplier effects and reduce inefficiencies within the industrial chain.

Second, the advancement dimension highlights the importance of upgrading toward high value-added, innovation-driven sectors. The forecasted increase in total output should not be interpreted as a mere continuation of quantitative growth, but rather as a signal that structural upgrading is necessary to sustain long-term competitiveness [57]. For example, investment in digital transformation, sports technology, and health-related services will be crucial to move the industry from labor-intensive production to knowledge-intensive and innovation-led development. This aligns with broader trends of industrial modernization observed in other emerging economies.

Third, the predicted growth also underscores the strategic coupling between the sports industry and national development goals. As the industry expands, it can provide strong support for the “Healthy China” and “Sports Power” initiatives by improving public health, stimulating domestic consumption, and creating new employment opportunities. This illustrates how the advancement of the sports industry is not only an economic process but also a social and institutional transformation, consistent with the integrated goals of high-quality development [58].

In the coming years, the growth rate of the sports industry is projected to surpass expectations, with new development opportunities emerging in sectors such as sports events, the sporting products industry, community sports, and youth sports. Investment trends are increasingly diversified, particularly in areas such as e-sports [59], sports technology [60], the outdoor economy [61], niche sports, and specialized sports education and training for women and youth [62]. Advances in digital transformation are further unlocking the potential of the sports technology sector. At the same time, government policy support continues to drive the expansion of sports facilities in both quantity and area, helping to reduce the urban-rural disparity in access to sports venues.

In summary, the forecasts derived from the GM (1,1) model gain explanatory depth when interpreted through the theoretical framework of industrial structure optimization. They suggest that China’s sports industry is at a critical juncture: while quantitative growth is expected to continue, achieving sustainable and high-quality development will require deeper structural adjustments, technological upgrading, and strategic alignment with broader national objectives.

### Limitations related to COVID-19

While the GM (1,1) model demonstrates promising performance in short-term forecasting with limited data, it also has inherent limitations. One key assumption of the GM (1,1) model is that the underlying data follow an exponential growth pattern [63]. This assumption may not hold in contexts where the system is approaching maturity, subject to significant policy interventions, or experiencing extraordinary external shocks [64]. In such scenarios, the divergence between the assumed exponential growth and the actual trajectory may compromise the model’s predictive accuracy. For instance, industries affected by market saturation, regulatory changes, or other exceptional events may exhibit growth dynamics that the GM (1,1) model cannot fully capture [65].

In the context of this study, the data encompass the COVID-19 pandemic years (2020–2022), during which sports participation, facility use, and industrial output were substantially disrupted. These unprecedented conditions represent an external shock that could have influenced the observed trends and introduced deviations from typical growth patterns. Consequently, the assumptions underlying the GM (1,1) model may be partially violated during this period, potentially affecting forecasting reliability. Furthermore, the model’s reliance on a single variable limits its ability to account for multifaceted influences, such as pandemic-related policy measures and consumer behavior changes [66]. These considerations highlight the importance of interpreting the results with caution and suggest that complementary or hybrid modeling approaches incorporating additional variables may enhance the robustness of the predictions.

## Implications

### Theoretical implications

This study contributes to the broader literature on industrial economics and development studies by demonstrating how industrial structure optimization theory can be applied to an emerging sector such as the sports industry. The integration of grey relational analysis with the GM (1,1) prediction model highlights the methodological value of grey system theory in examining complex industrial systems under uncertainty and limited data availability. Beyond the case of China, this research provides a replicable analytical framework for studying the dynamic interactions between sub-sectors and national economic development, thereby offering new insights into the structural evolution of industries in other emerging economies.

### Practical implications

From a practical perspective, the findings of this study have implications for sports practitioners and policymakers beyond China. First, identifying the key growth-driving sub-sectors underscores the importance of balancing manufacturing and service industries in order to foster sustainable development. This insight is relevant for countries that are currently in the process of sports industry modernization, such as those in Southeast Asia and Eastern Europe. Second, the forecasting results provide a reference for industry stakeholders to anticipate future demand trends, allocate resources more effectively, and design targeted investment strategies. Finally, by linking industrial development with broader social goals, such as public health, employment creation, and digital transformation, the study offers guidance for practitioners to align sports industry strategies with national development priorities across different contexts.

## Conclusion

This study provides a comprehensive analysis of China’s sports industry structural optimization through grey relational analysis and GM (1,1) prediction modeling, grounded in the political-economic context of socialist market economy. Results reveal that the manufacturing and sales sectors of sporting products and related products exhibit the strongest correlation with GDP, underscoring their critical role in driving economic growth. Furthermore, the GM (1,1) model effectively predicts future trends in the sports industry’s added value, offering insights for policymakers and industry stakeholders, such as recommendations for targeted investment and policy adjustments.

Despite providing detailed data analysis and model-based forecasts, this study has several limitations. First, it does not fully account for the effects of policy and environmental changes on the sports industry. External factors such as macroeconomic policies, regional development initiatives, socio-cultural trends, and environmental sustainability may substantially influence industry growth, distribution, and transformation, yet these variables were not explicitly incorporated into this study. Second, the study has limited interdisciplinary integration, with minimal application of theoretical frameworks or methodologies from economics, sociology, and management. This constrains a comprehensive understanding of the sports industry’s complexity, particularly regarding its interactions with socio-economic development and market dynamics.

To address these limitations, future research could adopt more targeted approaches. One direction is to incorporate policy interventions, regulatory shifts, and environmental factors into forecasting models, such as multi-variable GM (1,1), hybrid grey models, or system dynamics approaches, to improve predictive accuracy and adaptability under changing conditions. Another avenue is to enhance interdisciplinary analysis by integrating theories from economics (e.g., industrial upgrading), sociology (e.g., consumer behavior, social participation), and management (e.g., organizational strategy, innovation diffusion), providing a more nuanced understanding of the mechanisms driving sports industry development. Finally, future studies could complement quantitative modeling with qualitative and mixed-method research, such as case studies, interviews, and stakeholder surveys, to explore causal pathways, contextual factors, and practical implications, thereby validating and enriching the findings from quantitative analyses. These focused directions can guide subsequent research toward more actionable insights and a deeper understanding of China’s evolving sports industry.

In sum, this study offers essential theoretical support and practical guidance for the high-quality development of China’s sports industry. As the industry continues to grow and innovate, its impact on economic growth, public health, and social progress will become increasingly evident. Thus, continually optimizing the sports industry structure and fostering its synergy with the national economy are essential steps toward achieving sustainable development goals.

## Supporting information

S1 File Code. (ZIP)
